# Spontaneous Perirenal Hemorrhage Mimicking Renal Rupture in a Long‐Term Hemodialysis Patient: A Case Report

**DOI:** 10.1002/ccr3.72008

**Published:** 2026-02-06

**Authors:** Zhen Wang, Bo Yang, Yi Zhang, Xiaoqiang Li, Bin Wang, Jinghan Chen

**Affiliations:** ^1^ Department of Nephrology Shanghai Baoshan District Wusong Central Hospital (Wusong Branch, Zhongshan Hospital Affiliated to Fudan University) Shanghai China; ^2^ Department of Urology Shanghai Baoshan District Wusong Central Hospital (Wusong Branch, Zhongshan Hospital Affiliated to Fudan University) Shanghai China; ^3^ Department of General Surgery Shanghai Baoshan District Wusong Central Hospital (Wusong Branch, Zhongshan Hospital Affiliated to Fudan University) Shanghai China; ^4^ Department of Pathology Shanghai Baoshan District Wusong Central Hospital (Wusong Branch, Zhongshan Hospital Affiliated to Fudan University) Shanghai China; ^5^ Department of Radiology Shanghai Baoshan District Wusong Central Hospital (Wusong Branch, Zhongshan Hospital Affiliated to Fudan University) Shanghai China

**Keywords:** case report, hemodialysis, nephrectomy, renal artery embolization, spontaneous perirenal hemorrhage, spontaneous renal rupture

## Abstract

Spontaneous perirenal hemorrhage (SPH) and spontaneous renal rupture (SRR) are rare emergencies that can be radiologically indistinguishable when hematomas are large, especially in hemodialysis patients. We report a 53‐year‐old man on long‐term hemodialysis who developed acute left flank pain 19 h after low‐molecular‐weight heparin. Contrast‐enhanced CT suggested SRR without tumor. Urgent angiography revealed no active extravasation; empiric selective renal artery embolization was performed, but pain and anemia progressed (hemoglobin nadir 54 g/L), prompting emergency nephrectomy. Gross and microscopic pathology demonstrated an intact renal capsule with hemorrhage confined to perirenal fat and no neoplasm, cyst rupture, aneurysm, or vasculitis, confirming SPH. Uremia‐associated vasculopathy and repeated anticoagulation likely increased vascular fragility; bleeding from perirenal fat with collateral supply (lumbar, adrenal, gonadal arteries) may explain embolization failure. Postoperatively, dialysis anticoagulation was temporarily switched to nafamostat mesylate and later safely resumed with low‐molecular‐weight heparin; at approximately 7 months after surgery, hemoglobin was 109 g/L with no recurrence. This case underscores the limits of CT specificity in large hematomas, the need to consider extracapsular bleeding when embolization fails, the role of pathology for definitive diagnosis, and the importance of early surgical exploration and individualized anticoagulation in hemodialysis patients.

## Key Clinical Message

In long‐term hemodialysis, massive perirenal hematoma may reflect spontaneous perirenal hemorrhage without renal rupture; inconclusive imaging may require surgery and pathology. Failed renal artery embolization with instability suggests extracapsular bleeding from non‐renal collaterals, favoring early surgery. Individualized anticoagulation (nafamostat, then cautious LMWH) can safely continue dialysis.

## 1 Introduction

Spontaneous perirenal hemorrhage (SPH) and spontaneous renal rupture (SRR) are rare but potentially fatal emergencies [1, 2, 3]. Patients usually present with acute and severe symptoms, including sudden abdominal pain and hypotensive shock, which may indicate significant internal bleeding [4]. Computed tomography with intravenous contrast is the diagnostic mainstay, yet large hematomas can obscure the bleeding source and the status of the renal capsule, which complicates the distinction between SPH and SRR [5, 6]. Patients on maintenance hemodialysis are especially vulnerable because of uremic vasculopathy, vascular calcification, labile hypertension, and repeated anticoagulation; atrophic, cystic kidneys also confound imaging [7, 8, 9, 10]. Current care emphasizes rapid stabilization, selective renal artery embolization (SRAE) for stable patients with active extravasation, and surgery when embolization fails or malignancy is suspected [11, 12].

This case differs in three ways. First, CT suggested SRR in a patient receiving prolonged hemodialysis, yet pathology confirmed an intact capsule with hemorrhage confined to perirenal fat, which represents true SPH and is rarely documented in dialysis populations. Second, empiric SRAE failed, implicating extracapsular bleeding supplied by nonrenal collaterals such as lumbar, adrenal, and gonadal arteries, where embolization focused on the renal artery may be insufficient [13, 14]. Third, peridialysis anticoagulation was individualized with temporary nafamostat mesylate followed by resumption of low molecular weight heparin, consistent with emerging evidence for high bleeding risk settings [15]. Reporting this case clarifies the distinction between SPH and SRR, underscores the diagnostic limitations of CT, and supports early, multidisciplinary escalation from endovascular therapy to surgery in patients with end‐stage renal disease (ESRD). This case report has been reported in line with the SCARE checklist [16].

## 2 Case History/Examination

A 53‐year‐old male with a 6‐year history of maintenance hemodialysis presented to the emergency department with acute left flank pain lasting for 3 h on November 29, 2024. Before this visit, the patient's anticoagulant therapy was limited to low‐molecular‐weight heparin (LMWH) administered during hemodialysis sessions. During the hemodialysis session on November 28, 2024, nadroparin calcium (4100 IU) was administered for anticoagulation. Nineteen hours later, the patient developed acute left flank pain. There was no history of trauma. Nine years earlier, he was found to have proteinuria and elevated serum creatinine. He was diagnosed with chronic glomerulonephritis at that time, without a renal biopsy. His past medical history included stage 5 chronic kidney disease, well‐controlled hypertension for 8 years, and a left forearm arteriovenous fistula placed in December 2017. The patient reports no history of drug allergies, tobacco use, or alcohol consumption. There is no family history of similar conditions. Social and psychological functioning are intact. Upon examination, the patient was with a temperature of 36.6°C, a pulse of 90 beats per minute, a respiratory rate of 20 breaths per minute, and blood pressure of 121/105 mmHg. Left lumbar percussion tenderness; otherwise, the physical examination was unremarkable. He did not exhibit any symptoms of nausea, vomiting, or diarrhea.

## 3 Differential Diagnosis, Investigations and Treatment

**FIGURE 1:**
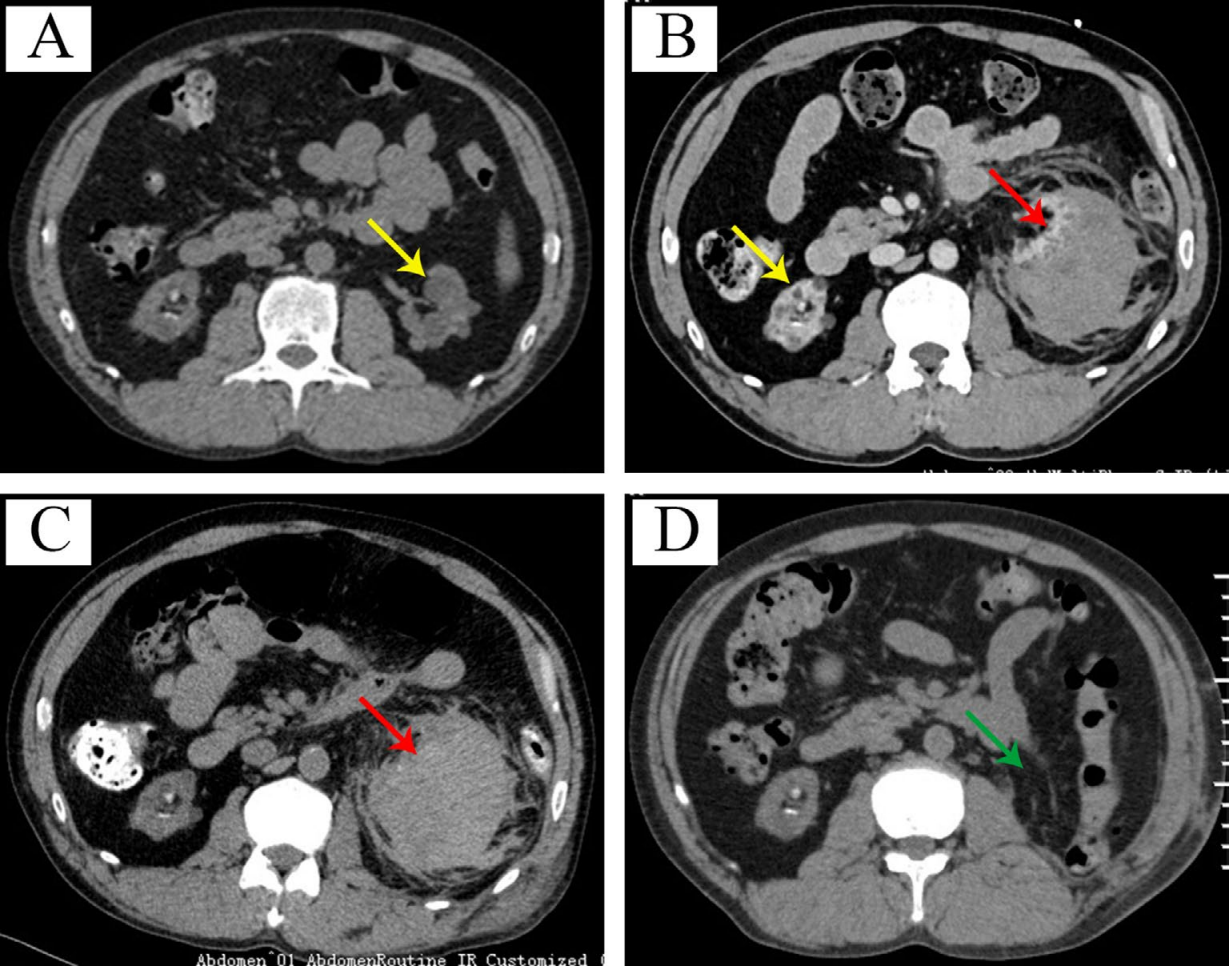
Series of CT examinations in a hemodialysis patient with spontaneous perirenal hemorrhage mimicking renal rupture. (A) CT scan dated April 7, 2024, shows bilateral kidney atrophy and multiple renal cysts (yellow arrow). A small area of hyperdensity surrounding the atrophic left kidney was considered more likely to represent an imaging artifact rather than true hemorrhage. (B) Enhanced CT performed on November 29, 2024, reveals a lobulated mixed‐density lesion in the left renal area, suggestive of a subcapsular hematoma due to suspected renal rupture (red arrow). (C) CT scan from December 2, 2024, presents a lobulated lesion with heterogeneous high and low density in the left renal area, indicative of renal rupture accompanied by hematoma formation, with increased perirenal fluid compared to the CT on November 29, 2024. (D) CT scan from January 8, 2025, shows the status post‐left nephrectomy (green arrow indicates the previous location of the left kidney).

Figure 1 demonstrates the morphological changes of the kidneys at different time points during the clinical course. Compared with the CT scan from the patient's routine physical examination on April 7, 2024 (Figure 1A), the CT scan on November 29, 2024 (Figure 1B) revealed an irregular mass in the left renal region, suggestive of SRR. As shown in Figure 1B, the contrast‐enhanced CT scan performed at that time demonstrated no enhancement at the lesion site, effectively ruling out a tumor. The imaging also demonstrated significant perirenal fluid accumulation and thickening of the renal fascia. In addition, the right kidney showed signs of atrophy and multiple calcifications. Laboratory findings indicated anemia, with a hemoglobin level of 102 g/L and a platelet count of 146 × 10^9^/L. Given the critical condition, the patient was urgently hospitalized on November 29, 2024. Following admission, the patient received bed rest, hemostatic therapy, anti‐inflammatory treatment, and nutritional support.

**FIGURE 2:**
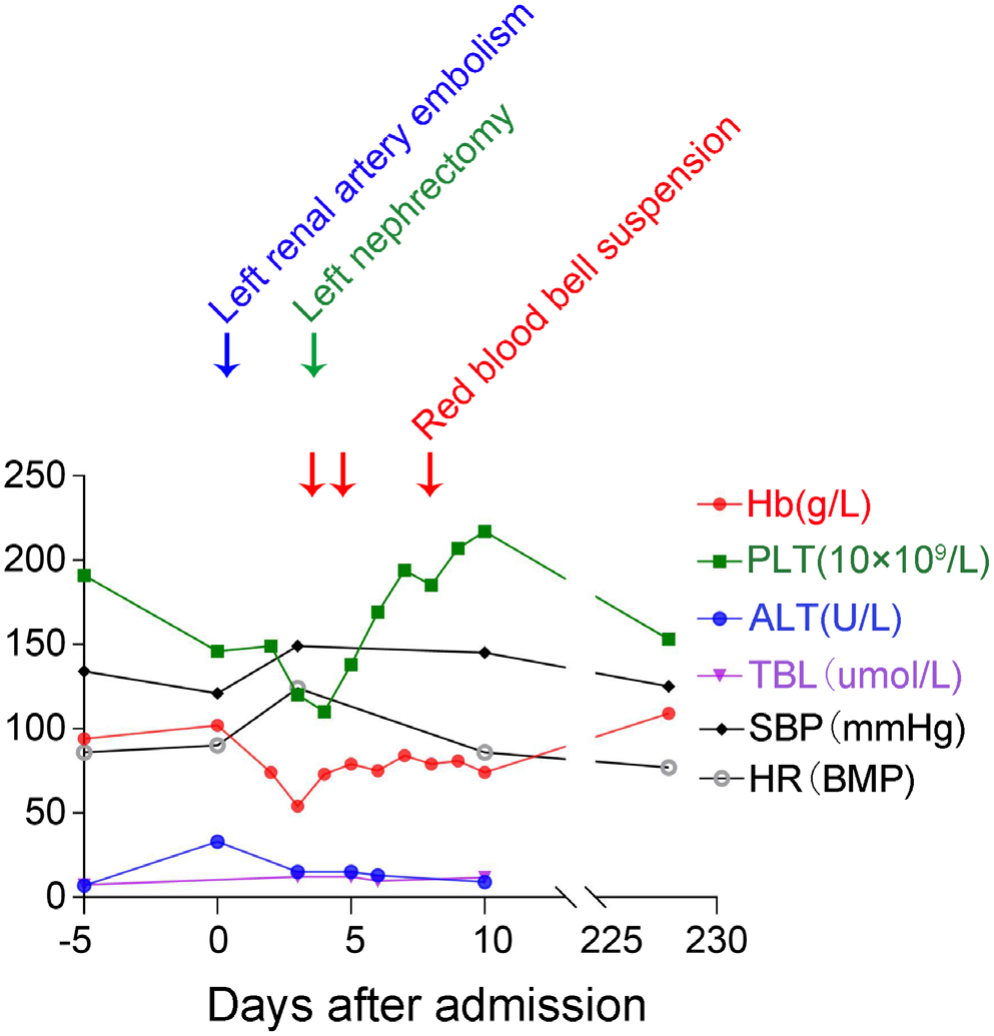
Changes in selected laboratory parameters and major interventions in a hemodialysis patient with spontaneous perirenal hemorrhage mimicking renal rupture. The graph illustrates the variations in hemoglobin (Hb, g/L), platelet count (PLT, ×10^9^/L), alanine aminotransferase (ALT, U/L), and total bilirubin (TBL, μmol/L) over time. Key clinical events are indicated: left renal artery embolism (blue arrow), left nephrectomy (green arrow), and red blood cell suspension transfusions (2 units on days 3 and 4 and 1 unit on day 7 after admission, indicated by red arrows). Notably, following the left nephrectomy, the patient's condition stabilized, and hemoglobin levels improved. BMP, beats per minute; HR, heart rate; SBP, systolic blood pressure.

**FIGURE 3:**
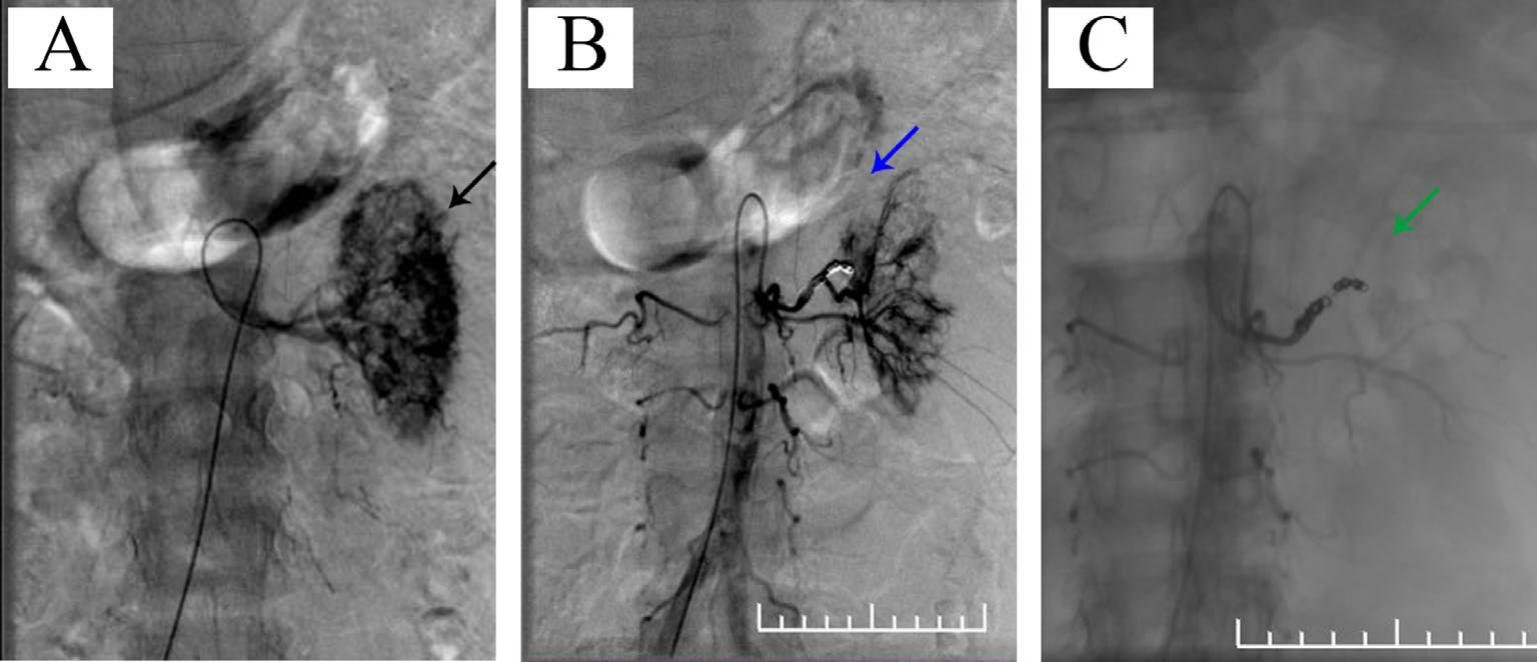
Angiographic findings in a patient with suspected renal rupture later proven to have spontaneous perirenal hemorrhage. (A) Selective left renal arteriogram showing a suspected bleeding focus in an upper‐pole branch with subtle pooling of contrast (black arrow). (B) After a microguidewire and microcatheter were advanced into a distal bifurcation near the superior division of a second‐order branch, two 2 mm × 6 mm detachable coils were deployed. The microcatheter was then withdrawn to the proximal main left renal artery near the bifurcation, where one 3 mm × 6 mm and one 3 mm × 12 mm detachable coil were placed; the corresponding branches are no longer opacified (blue arrow). (C) Completion angiogram after additional embolization of the left renal artery with 1000‐μm gelatin sponge particles, demonstrating markedly reduced renal arterial flow (green arrow).

Figure 2 shows the changes in the patient's key laboratory parameters (hemoglobin, platelet count, alanine aminotransferase, and total bilirubin) at various time points during hospitalization, along with the timing of major clinical interventions. On the day of admission (Day 0), the patient underwent renal angiography and left selective renal artery embolization (SRAE) under local anesthesia (Figure 3). Although no definite high‐flow source of hemorrhage, such as pseudoaneurysm formation or active extravasation, was identified, subtle contrast pooling in an upper‐pole branch suggested possible slow bleeding. Given CT findings suggestive of renal rupture, a large perirenal hematoma, persistent flank pain, and a falling hemoglobin level in a patient already on long‐term hemodialysis, empiric renal‐artery embolization was therefore performed: A microcatheter was advanced into a second‐order superior branch, where two 2 × 6 mm coils (0.018‐in. system) were deployed, followed by one 3 × 6 mm and one 3 × 12 mm coil in the proximal main left renal artery and additional embolization with 1000‐μm gelatin sponge particles. Despite these interventions, the patient's blood pressure remained relatively stable without a significant drop. However, by the third day of hospitalization, his acute left flank pain and tachycardia had progressed, with a heart rate of up to 124 beats per minute. His hemoglobin level had decreased to 54 g/L, while the platelet count remained at 120 × 10^9^/L (Figure 2). Considering the patient's deteriorating condition and the suspicion of ongoing bleeding, a multidisciplinary discussion was organized that included specialists in urology, general surgery, hematology, and radiology. Repeat angiography focusing on potential collateral sources (including lumbar, adrenal, and gonadal arteries) was considered. However, since the left SRAE had failed to control the hemorrhage and the patient remained hemodynamically unstable, perirenal bleeding was suspected; therefore, our team decided to proceed with emergency surgery.

An emergency left nephrectomy was performed under general anesthesia on Day 3. Throughout the hospitalization, including the period immediately before nephrectomy, there were no significant abnormalities noted in serum glutamyl pyruvate transaminase, total bilirubin, thrombin time (TT), or prothrombin time (PT), and platelet counts remained within the normal range. The indication for emergency surgery was therefore driven by progressive anemia and clinical deterioration rather than correction of a specific coagulopathy. Thromboelastography (TEG) was not performed because it is not routinely available in our institution for emergency surgical cases. During the operation, a perirenal hematoma was observed on the left side, with significant blood accumulation in the perirenal fat and unclear delineation between the perirenal fascia and renal capsule. A complete resection of the left kidney and surrounding fatty tissue was performed. All interventional and surgical physicians participating in the rescue treatment were senior attending physicians.

**FIGURE 4:**
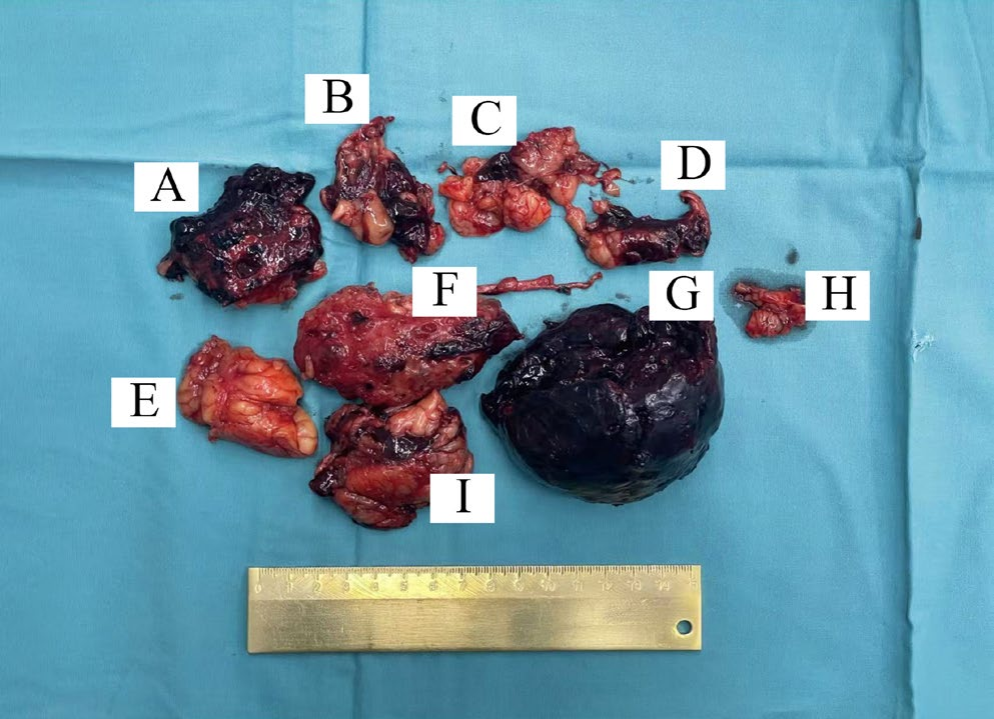
Gross specimen confirming spontaneous perirenal hemorrhage without renal rupture. The left kidney weighs 50 g and measures 7.4 × 3.9 × 4.1 cm (F). It is free of the perirenal capsule, with a smooth and intact surface, showing no evidence of rupture. The renal parenchyma thickness varies from 0.5 to 1.2 cm and contains multiple small cysts. A 4.1 cm long and 0.4 cm in diameter remnant of the ureter is also noted. Additionally, there is a dark red, soft, hemispherical blood clot tissue measuring 9.4 × 6.9 × 6.7 cm (G), consistent with perirenal hemorrhage. Additionally, fragments of perirenal adipose tissue (Images A–E, H, I) exhibit evidence of bleeding.

SPH can be caused by the rupture of renal tumors, cysts, and other underlying etiologies [10, 11]. The left kidney (Figure 4F) weighed 50 g and measured 7.4 × 3.9 × 4.1 cm, noticeably smaller than normal. The renal capsule was completely intact and smooth, without any fissures or tear marks. The parenchyma was thinned (0.5–1.2 cm thick), and multiple small cortical cysts were observed. No aneurysms or thrombi were present in the vessels. Dark red, hemispherical blood clots (Figure 4G, 9.4 × 6.9 × 6.7 cm) were found completely separated from the kidney, located outside the renal capsule, and were not continuous with the renal cortex. Additionally, multiple perirenal fat masses (Figure 4A–E,H,I) demonstrated infiltrative hemorrhage, indicating that the true source of bleeding was within the perirenal fat rather than the renal parenchyma or subcapsular space.

**FIGURE 5:**
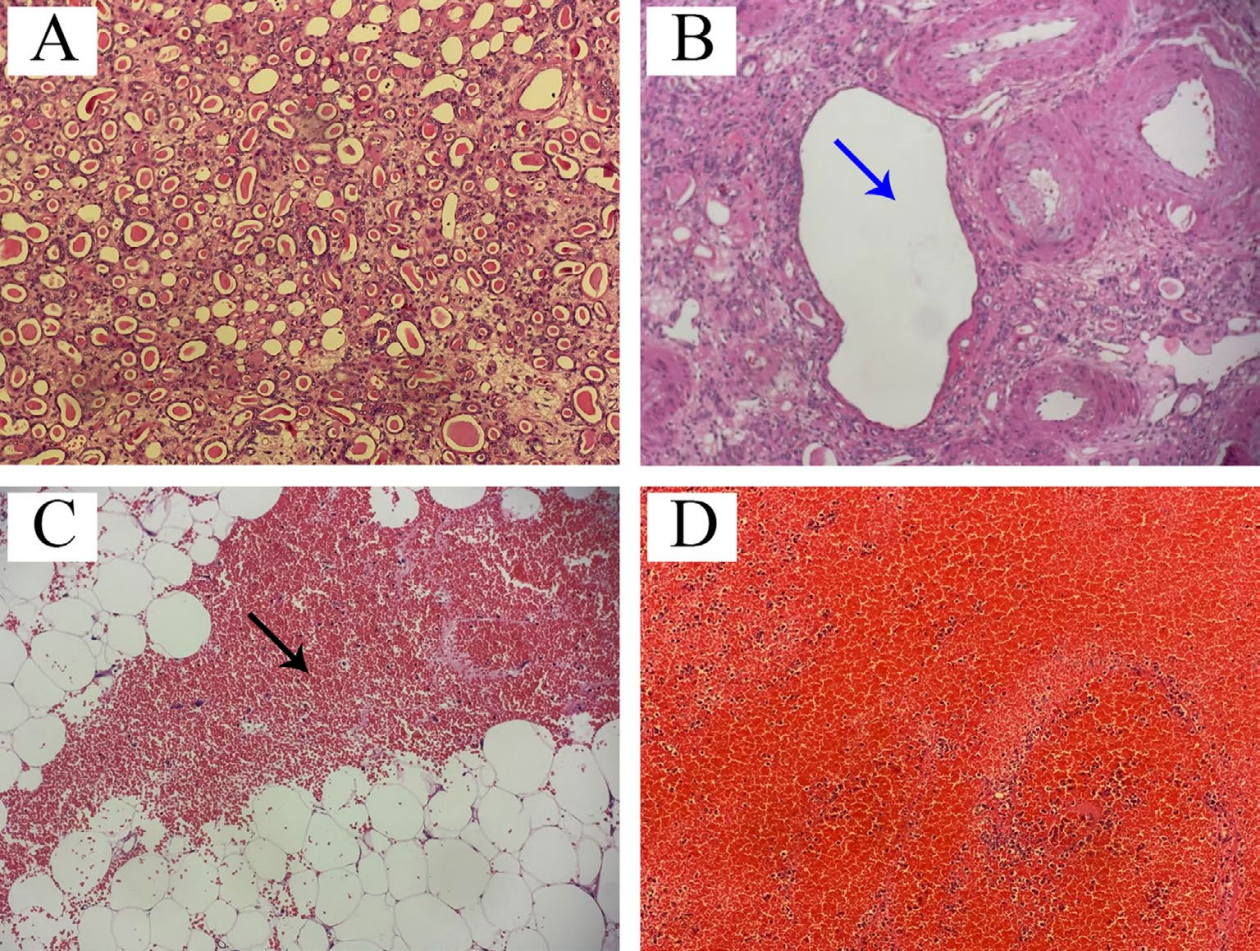
Histopathological findings of resected tissue confirming spontaneous perirenal hemorrhage (Hematoxylin–eosin staining, ×200). (A) This section predominantly displays the renal tubules. (B) Renal cysts are observed (indicated by blue arrows). (C) a region of hemorrhage within the adipose tissue (shown by the black arrow), indicating a focal area of bleeding and inflammatory response. (D) The pathological structure of the blood clot is characterized by a prominent red coloration, reflecting the abundance of red blood cells. Among these, images (A) and (B) are derived from the excised right kidney (Figure 4F), image (C) is from the bleeding adipose tissue/fat sac (Figure 4A), and image (D) is from the blood clot (Figure 4G).

Microscopically, the renal parenchyma (Figure 5A) showed diffuse tubular atrophy, but no evidence of necrosis or vasculitic changes. The cortical cyst (Figure 5B) had an intact wall without rupture or hemorrhage, and no tumor cells were observed. Small arteries within the renal tissue exhibit wall thickening and fibrosis without evidence of aneurysm formation. The perirenal fat (Figure 5C) contained extensive fresh hemorrhage dispersed among adipocytes, accompanied by mild infiltration of neutrophils and macrophages. The blood clot (Figure 5D) was composed predominantly of densely packed red blood cells with fibrin, consistent with a recent hematoma.

In summary, pathological examination confirmed an intact renal capsule and absence of cortical rupture, effectively ruling out SRR. The hemorrhage was strictly confined to the perirenal fat outside the capsule, consistent with SPH. There was no evidence of renal neoplasms, angiomyolipoma, or vasculitis. All cortical cysts remained intact. Notably, arterial wall thickening and fibrosis indicated that chronic uremia‐associated vasculopathy and repeated heparinization during hemodialysis, both of which increase vascular fragility, were the most likely predisposing factors for SPH in this patient.

## 4 Outcome and Follow‐Up

After surgery, the patient was transferred to the intensive care unit (ICU) for close monitoring. He received supportive care, including blood transfusions (Figure 2) and kidney replacement therapy, and was closely monitored for potential complications such as infection and electrolyte imbalances. In total, he received 5 units of packed red blood cells during hospitalization (2 units on hospital days 3 and 4, and 1 unit on day 7; Figure 2 and Table 2). Throughout the hospitalization, there were no significant abnormalities noted in serum glutamyl pyruvate transaminase, total bilirubin, thrombin time (TT), or prothrombin time (PT).

By day 10, the patient's hemoglobin level had risen to 74 g/L, he reported no abdominal pain, and a physical examination indicated stable vital signs and satisfactory healing of the surgical wound. Consequently, he was discharged from the hospital. The patient expressed great satisfaction with his hospitalization and thanked the medical staff for promptly treating his critical condition. Following discharge, the patient continued with routine hemodialysis three times a week. After the patient was diagnosed with bleeding, anticoagulation during hemodialysis was switched to nafamostat mesylate (150 mg per session), which was a valid alternative to citrate for hemodialysis patients at high risk of bleeding [15]. Approximately 2 months after nephrectomy, LMWH was reintroduced for anticoagulation during each hemodialysis session. This decision was made after all of the following conditions had been met: absence of any clinical evidence of ongoing bleeding, stable hemoglobin levels without further transfusion, complete healing of the surgical wound, and confirmation of no residual hematoma on follow‐up CT at POD 37. LMWH (nadroparin calcium 4100 IU at the start of each hemodialysis session) was resumed at the full standard dose without step‐wise up‐titration, under close clinical and laboratory monitoring, and no recurrent bleeding occurred during more than 5 months of follow‐up. At the latest follow‐up (approximately 7 months after surgery), his hemoglobin level had improved to 109 g/L, and he reported no discomfort. The patient's treatment involved multidisciplinary collaboration (Table 1) among general surgery, radiology, interventional medicine, hematology, urology, and pathology, which was essential for achieving a clear diagnosis and successful outcome. Table 2 summarizes the patient's treatment timeline.

**TABLE 1:** Contributions of the multidisciplinary team in the management of the patient.

Specialty	Specific contribution	Primary action
General Surgery	Upon admission, the General Surgery team identified acute symptoms and signs of ongoing bleeding, prioritized prompt intervention, and assessed surgical risk in the context of end‐stage renal disease (ESRD). Recognizing the complexity and high risk of surgery in a hemodialysis patient, they advocated for early multidisciplinary collaboration to ensure optimal patient care	Urged urgent multidisciplinary intervention
Radiology	Radiology and Interventional Medicine were consulted early. They performed contrast‐enhanced CT and diagnostic renal angiography to localize the source of hemorrhage, then carried out embolization to achieve minimally invasive hemostasis	Localized bleeding and performed embolization
Hematology	The Hematology team, in collaboration with the Nephrology/Dialysis team, recommended discontinuing low‐molecular‐weight heparin during dialysis and switching to nafamostat mesylate for anticoagulation to reduce bleeding risk. They also provided guidance on hemostatic management and blood transfusion, and supervised the monitoring of postoperative anemia and bleeding tendency	Adjusted dialysis anticoagulation and guided transfusion
Urology	Despite these interventions, the patient continued to experience significant bleeding and clinical deterioration. Given this ongoing instability, the Urology team, with input from the multidisciplinary team (including reassessment of operative risk by General Surgery and Hematology), decided to proceed with nephrectomy. Urology developed and coordinated the perioperative strategy, focusing on coagulation management (as advised by Hematology) and optimizing hemodynamic support in collaboration with Anesthesiology and Nephrology	Led nephrectomy with multidisciplinary input
Pathology	The pathological results showed an intact renal capsule without cortical rupture, ruling out SRR. The hemorrhage was confined to the perirenal fat, consistent with SPH	The diagnosis of SPH was confirmed.

**TABLE 2:** Timeline of events in chronological order.

Date	Hospital day	Event/intervention	Key findings/outcomes
07/04/2024	−236	Routine CT	Bilateral renal atrophy with multiple cysts; no definite hemorrhage
28/11/2024	−1	Hemodialysis with nadroparin calcium 4100 IU	Last dialysis session before symptom onset
29/11/2024	0	Acute left flank pain; and admission	Contrast‐enhanced CT suggested SRR; no tumor enhancement
29/11/2024	0	Renal angiography and selective left renal artery embolization	No definite extravasation or pseudoaneurysm; distal branch empirically embolized
29/11/2024	0	Anticoagulation during dialysis	Switched to nafamostat mesylate 150 mg per session
01–02/12/2024	2–3	Clinical deterioration and MDT discussion	Worsening pain and tachycardia; hemoglobin fell to 54 g/L
02/12/2024	3	Emergency left nephrectomy with evacuation of perirenal hematoma	Postoperative ICU monitoring; subsequent pathology confirmed intact renal capsule and perirenal fat hemorrhage consistent with SPH
02/12/2024	3	Red blood cell transfusion	2 units
03/12/2024	4	Red blood cell transfusiontransfusion	2 units
06/12/2024	7	Red blood cell transfusion	1 unit
09/12/2024	10	Discharge	Hemoglobin 74 g/L; vitals stable; wound healing satisfactory
08/01/2025	POD 37	Follow‐up CT	Post‐left nephrectomy status confirmed
11/02/2025	POD 71	Anticoagulation adjustment	Resumed LMWH for hemodialysis
15/07/2025	POD 225	Outpatient follow‐up	Hemoglobin 109 g/L; asymptomatic; no recurrence

Abbreviations: MDT, multidisciplinary team; POD, postoperative day (after left nephrectomy).

## 5 Discussion

We present a rare case of SPH in a 53‐year‐old man on long‐term hemodialysis, initially misdiagnosed as SRR based on CT imaging. Despite SRAE, bleeding persisted due to extracapsular hemorrhage supplied by nonrenal collaterals. Emergency nephrectomy was required, and pathological examination confirmed an intact renal capsule with hemorrhage confined to the perirenal fat—a presentation rarely documented in patients on dialysis. This case is unusual for three reasons: (1) SPH mimicked SRR on imaging in an atrophic dialysis kidney; (2) SRAE failed because bleeding originated from perirenal fat supplied by lumbar, adrenal, and gonadal arteries rather than the renal artery; and (3) pathological confirmation was essential for definitive diagnosis.

Distinguishing between SRR and SPH can be challenging, as both conditions may present with similar clinical symptoms, including acute abdominal pain and signs of shock [11, 17, 18]. The key anatomical distinction is that SRR refers to hemorrhage confined within the renal parenchyma or subcapsular space, whereas SPH denotes bleeding into the perirenal (extracapsular) space posterior to the peritoneum [4] Both may coexist, especially if a tumor or vascular lesion causes parenchymal rupture with perirenal extension [19]. Contrast‐enhanced CT is the primary diagnostic tool [12, 17, 18]. However, large hematomas can obscure the bleeding source, limiting CT specificity [4]. In our case, CT suggested SRR in a long‐term hemodialysis patient, but surgical and pathological findings ultimately demonstrated an intact renal capsule with bleeding confined to perirenal fat, confirming pure SPH. Angiography, MRI, and ultrasound can provide complementary information in selected patients [18, 20, 21]. However, in unstable patients, there is often limited time for extensive imaging, and definitive diagnosis may depend on surgical exploration and pathology.

Treatment strategies for SRR and SPH vary based on the patient's hemodynamic stability and the underlying cause [11]. In hemodynamically stable patients with minimal bleeding, conservative management may be sufficient, including observation and supportive care. For those with significant hemorrhage, SRAE is often preferred, as it is less invasive and can effectively control bleeding [22, 23]. For patients with a solitary kidney or severely impaired renal function, SRAE is widely regarded as the first‐line treatment, as it can be repeated to preserve remaining nephrons [24]. In many cases, surgical exploration and nephrectomy are required to manage rupture and control hemorrhage; these interventions are typically reserved for situations where SRAE fails or malignancy is suspected [4, 11, 25]. In the present case, given the initial clinical suspicion of SRR, SRAE was performed. As the patient was receiving maintenance hemodialysis and had no requirement for preservation of residual renal function, embolization was undertaken on the left renal artery. However, following the procedure, the patient's left flank pain worsened and the hemoglobin level declined further. Follow‐up CT imaging demonstrated ongoing active bleeding. After multidisciplinary consultation, perirenal bleeding was suspected, and immediate surgical intervention was undertaken. This surgical approach allowed for direct observation and treatment of the hematoma, ultimately leading to symptom relief and stabilization of the patient's condition.

The etiology of SPH in dialysis patients is multifactorial, with common causes including acquired cystic kidney disease, renal tumors, amyloidosis, trauma, anticoagulation during dialysis, and vascular disease [1, 10, 11, 19]. In this case, there was no evidence of kidney tumors, ruptured acquired renal cysts, uncontrolled hypertension, or trauma. Additionally, the hemorrhage did not originate from the renal parenchyma but occurred within the perirenal adipose tissue. Vascular lesions are more common in patients undergoing long‐term dialysis [10]. Chest CT in this patient revealed aortic and coronary artery sclerosis. We speculate that the patient's SPH is related to underlying vascular disease and the use of anticoagulation during hemodialysis. Although the patient was initially treated with SRAE, effective hemostasis was achieved only after resection of the kidney and perirenal tissue. This can be explained by the vascular supply of the perirenal region: while some blood vessels originate from the renal artery, there is also significant collateral perfusion from the lumbar, adrenal, and gonadal arteries [13, 14].

In our practice, switching from nafamostat back to LMWH during maintenance hemodialysis is considered only when all of the following conditions are met: absence of clinical signs of ongoing or recurrent hemorrhage, stable hemoglobin levels without the need for transfusion, satisfactory wound healing, and imaging confirmation of hematoma resolution, followed by multidisciplinary agreement that the bleeding risk has returned to baseline. In the present case, these criteria were met by POD 71, at which time LMWH was reintroduced at the usual full dose, without step‐wise escalation, under close monitoring and with no subsequent bleeding events.

This case report enriches the limited literature on SPH in hemodialysis patients by providing rare pathological evidence that massive perirenal bleeding can occur without parenchymal rupture. It illustrates the diagnostic challenges of distinguishing SRR from SPH using CT and renal artery embolization. It underscores the value of a multidisciplinary approach involving urology, surgery, hematology, and radiology. It also highlights improved postoperative clinical and hematologic outcomes in this vulnerable hemodialysis population. However, the report has limitations, including initial challenges in accurately identifying the bleeding site, which may indicate potential diagnostic inaccuracies. No additional imaging modalities, such as MRI or ultrasound, were utilized for comparative or supplementary diagnostic purposes in this case. Another limitation is that thromboelastography was not available in our institution, so perioperative coagulation management relied on conventional laboratory parameters rather than viscoelastic testing. Additionally, as with any case report, biases in case selection and interpretation could affect the objectivity of the conclusions drawn.

## 6 Conclusions

In conclusion, this case underscores the diagnostic and therapeutic challenges associated with SRR and SPH in hemodialysis patients. Because SPH can closely mimic SRR in both clinical presentation and radiological findings, pathological evidence is essential for accurate diagnosis. Multidisciplinary collaboration between nephrology, radiology, and urology is essential to ensure optimal diagnostic accuracy and patient outcomes. Careful differentiation between SPH and SRR is crucial for guiding individualized management strategies in this complex patient population.

## Author Contributions

**Zhen Wang:** conceptualization, data curation, funding acquisition, writing – original draft, writing – review and editing. **Bo Yang:** investigation, project administration. **Yi Zhang:** methodology, project administration. **Xiaoqiang Li:** investigation, methodology. **Bin Wang:** investigation, methodology, writing – original draft. **Jinghan Chen:** funding acquisition, investigation, methodology, writing – review and editing.

## Funding

This work was supported in part by funding from the Shanghai Baoshan District Medical Key Specialty Project (BSZK‐2023‐BP03), the Baoshan District Science Popularization Project of Shanghai (1‐L007), and the Medical and Health Project of the Shanghai Baoshan Science and Technology Commission (2023‐E‐06).

## Ethics Statement

This study adhered to the principles of the Declaration of Helsinki and received approval from the Ethics Committee of Wusong Central Hospital, Baoshan District, Shanghai (Project Number: 2025‐P‐2).

## Consent

Written informed consent for publication of his clinical details and clinical images was obtained from the patient.

## Conflicts of Interest

The authors declare no conflicts of interest.

## Data Availability

The original contributions presented in this study are included within the article. Further inquiries may be directed to the corresponding author.
